# Impact of clinical urgency, physician supply and procedural capacity on regional variations in wait times for coronary angiography

**DOI:** 10.1186/1472-6963-10-5

**Published:** 2010-01-05

**Authors:** Harindra C Wijeysundera, Therese A Stukel, Alice Chong, Madhu K Natarajan, David A Alter

**Affiliations:** 1 Division of Cardiology, Schulich Heart Centre and Department of Medicine, Sunnybrook Health Sciences Centre, University of Toronto, Ontario, Canada; 2 Institute for Clinical Evaluative Sciences, Toronto, Ontario, Canada; 3 Hamilton Health Sciences, McMaster University, Ontario, Canada; 4 The Li Ka Shing Knowledge Institute of St. Michael's Hospital, University of Toronto, Ontario, Canada; 5 The Cardiac Prevention Program of the Toronto Rehabilitation Institute, Ontario, Canada

## Abstract

**Background:**

Despite universal health care, there continues to be regional access disparities to coronary angiography in Canada. Our objective was to evaluate the extent to which demand-side factors such as clinical urgency/need, and supply-side factors, as reflected by differences in physician and procedural supply account for these inequalities.

**Methods:**

Our cohort consisted of 74,254 consecutive patients referred for coronary angiography in Ontario, Canada between April 1^st ^2005 and March 31^st ^2006, divided into three urgency strata based on a clinical urgency scale. Cox-proportional hazard models were developed, adjusting for age, gender, socioeconomic status (SES), region, and urgency score, with greater hazard ratios (HR) indicating shorter wait times. To evaluate mediators of any residual wait-time differences, we examined the influence of the regional supply of cath lab facilities, invasive cardiologists and general practitioners (GP).

**Results:**

We found that the urgency score was a significant predictor of wait time in all three strata (urgent patients: HR 1.61 for each unit increase in patient urgency (95% Confidence interval (CI) 1.55-1.67); semi-urgent patients: HR 1.55 (95% CI 1.44-1.68); elective patients: HR 1.13 (95% CI 1.08-1.18)). After accounting for clinical need/urgency, regional wait time differences persisted; these were most consistently associated with variation in cath lab supply. The impact of invasive cardiologist supply was restricted to urgent patients while that of GP supply was confined to semi-urgent and elective patients.

**Conclusion:**

We found that there remained significant regional disparities in access to coronary angiography after accounting for clinical need. These disparities are partially explained by variations in supply of both procedural capacity and physician services, most notably in elective and semi-urgent patients.

## Background

Canada has a single payer universal health care system, one goal of which is equal access to medical services for all citizens. Since its implementation, there has been substantial evidence of improved access to care[1]. Nonetheless, there continues to be access disparities in the utilization of coronary angiography; these are driven by socioeconomic status (SES), geographical proximity to tertiary care facilities and the availability of on-site procedural capacity[2-5]. As such, explicit prioritization criteria predicated on patient need have been advocated by policy makers as a more appropriate means to triage patients awaiting coronary angiography[6-8]. However, the effectiveness of such demand-side wait-time management tools in addressing access inequalities remains uncertain[9,10].

The Cardiac Care Network (CCN) is a centralized, province-wide registry of patients waiting for coronary angiography, angioplasty and bypass surgery in the province of Ontario[11]. In support of this registry, a 1993 Canadian physician expert panel developed an explicit urgency rating scale, based on five clinical parameters, to be used for coronary angiography triage. In addition, recommended maximum waiting times (RMWT) were allocated based on urgency scores[12]. This urgency score has been validated as an accurate measure of clinical need given its correlation with implicit physician judgment and the risk of adverse events in patients on the waiting list[13]. In 2000, the CCN adopted and implemented the explicit angiography rating scale as a system monitoring and surveillance tool to help institutions triage their patients for coronary angiography and facilitate adherence to RMWT.

In contrast to demand-side initiatives to managing wait-times, supply-side strategies to improve hospital capacity and work force have been the dominant approach to addressing wait-time inequalities[14]. For coronary angiography, three supply measures are of relevance. Given that the family practice physician/general practitioner (GP) is the portal of entry into the health care system for the majority of patients, GP supply is a reflection of overall access to the health care system. The regional capacity to perform coronary angiography is measured by both the available workforce, as reflected in the supply of invasive cardiologists, and by the overall infrastructure, as reflected in supply of cath labs.

Accordingly, the objective of the present study was to evaluate if access inequalities, measured by wait time for coronary angiography, across geographic regions persist after accounting for demand-side factors, such as age, gender, SES and clinical need, as defined by an explicit urgency score. Furthermore, we explored the contribution of regional supply differences to any residual waiting times disparities after adjusting for demand-side factors.

## Methods

### Local Health Networks

**Figure 1:**
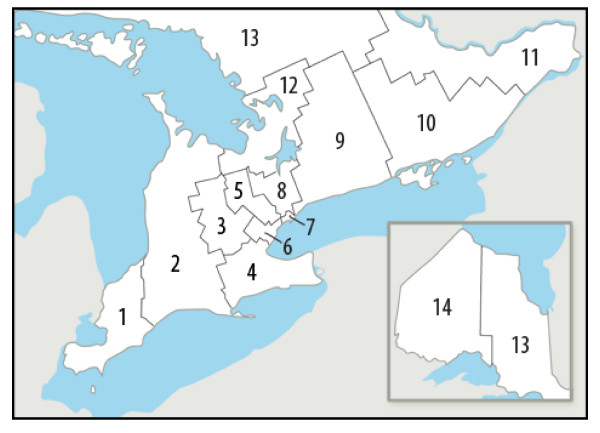
**Regional Local Health Integration Networks (LHIN) in Ontario**. 1 Erie St. Clair. 2 South West. 3 Waterloo Wellington. 4 Hamilton Niagara Haldimand Brant. 5 Central West. 6 Mississauga Halton. 7 Toronto Central. 8 Central. 9 Central East. 10 South East. 11 Champlain. 12 North Simcoe Muskoka. 13 North East. 14 North West.

The Ontario Ministry of Health and Long-term Care funds angiography slots on a per-capita basis to tertiary care centers in order to service their catchment area[15]. Although no predefined referral networks exist, tertiary centers typically service their surrounding region[16]. In 2006, the Ontario Ministry of Health and Long-Term Care transferred the responsibility for planning, integrating and funding of health services within the province to 14 regional Local Health Integration Networks (LHIN)(Figure 1). The boundaries of each LHIN were used to assess any geographic inequalities in access to coronary angiography.

### Data Sources

Referral information was obtained from the CCN provincial registry which prospectively tracks the use of coronary angiography and cardiac revascularization procedures throughout the province. The provincial referral form includes data on age, gender, patient location, Canadian Cardiovascular Society (CCS) angina classification, baseline ST-segment shifts, noninvasive cardiac testing, recent myocardial infarction, co-morbidity and concomitant selected pharmaco-therapies. Once a referral form is received by an institution, the patient is prioritized for angiography with the urgency score used as a management tool. Wait times were determined from the time that the CCN referral form was completed by the referring physician to the time that the coronary angiogram was completed.

The present study consists of consecutive patient encounters on the waiting list for coronary angiography for the fiscal year of 2005 to 2006. We only included patients who were residents of the province of Ontario and had a valid health card number who were undergoing coronary angiography (n = 113,313). For patients with repeat angiograms, we included only the first procedure in our analysis (n = 84,882). We excluded patients in whom data on age, sex, postal code (used to derive LHIN and income), referring cardiologist, wait time, RMWT, referral date, or urgency score was missing (n = 10,628). Finally, we also excluded patients with ST-segment elevation myocardial infarctions, who underwent primary angioplasty or rescue angioplasty as the CCN referral form is not completed prior to the procedure in these patients.

### Coronary Angiography Urgency Score

The urgency score comprises five clinical measures: CCS angina classification, the presence or absence of resting or dynamic ST segment changes; the presence or absence of early positive exercise-induced ischemia on treadmill testing; the presence or absence of high-risk reversible ischemia on myocardial perfusion testing (anterior or multi-vessel coronary ischemia); and the presence or absence of recent myocardial infarction (12 weeks of less)[12]. Based on presenting symptoms, each patient was assigned a urgency score ranging from 1 to 7, with 7 being the most urgent[12]. The urgency score has intervals of 0.1 and is evaluated as a continuous variable. Each urgency score corresponded to RMWT. For the purpose of our analysis, patients were stratified into three levels of urgency: urgent (4.0 ≤ urgency score ≤ 7.0; RMWT ≤ 7 days), semi-urgent (3.1 ≤ urgency score ≤ 3.9; 7 days < RMWT < 13 days), and elective (1.0 ≤ urgency score ≤ 3.0; RMWT > 14 days)[12].

### Socioeconomic Status and Supply Measures

We used postal codes to determine the Canadian census neighborhood income quintile. Regional supply measures included regional allocation of cath lab facilities (both with and without on-site surgical back-up), and invasive cardiologists (both with and without angioplasty skills) per 100,000 persons within the LHIN. GP supply was per 10,000 persons within the LHIN. Information on regional physician supply was obtained from the Ministry of Health and Long-term Health billing database.

### Statistical Analyses

A 2-level hierarchical Cox-proportional hazards model was developed with the time to coronary angiography as the response variable. As such, a hazard ratio (HR) greater than one would indicate a shorter wait time for angiography. Our hierarchical model clustered by the referring physician, because patients referred by the same physician may have had correlated outcomes, leading to an underestimation of standard errors; we included a working correlation matrix in the estimation procedure to adjust the standard errors appropriately. Models were stratified into elective, semi-urgent and urgent categories. The co-variates of interest were the urgency score, age, gender, income, and LHIN. Age and gender were reclassified into age-gender specific categories of 20-39 years, 40-64 years, 65-74 year and greater than 75 years.

In order to evaluate the influence of regional supply measures for geographic differences between LHINs, age-gender and urgency score adjusted models were developed substituting LHIN with cath lab, invasive cardiologist and GP supply.

As a sensitivity analysis, we repeated our analysis in an earlier cohort using patient encounters from 2001 to 2004 to explore any differences between periods. These results are found in Additional File 1.

All statistical analyses were performed with the SAS statistical software (version 9.1, SAS Institute Inc, USA). Permission for access to CCN data was granted by the CCN research and publications committee. This study was approved by the Research Ethics Board at Sunnybrook Health Sciences Centre.

## Results

The cohort consisted of 74,254 patients, of whom 28,680 were urgent, 31,545 were semi-urgent and 14,029 were elective in nature (Table 1). The majority of patients were between the ages of 40 to 64 years with women representing approximately one third of the cohort. The patients were distributed evenly across the five income quintiles, irrespective of urgency category. As expected, urgent patients had a mean wait time (2.4 days) that was substantially shorter than that of the semi-urgent or elective patients (13.1 days and 17.3 days respectively).

**Table 1:** Baseline Characteristics of Cohort, Stratified by Urgency Category

Variable		Urgent	Semi-urgent	Elective
N		28,680	31,545	14,029
				
age	*mean +/- sd*	63.9 +/- 12.8	64.3 +/- 11.4	61.0 +/- 10.8
age group	*20-39*	777 (2.7%)	519 (1.7%)	309 (2.2%)
	*40-64*	13,694 (47.8%)	14,997 (47.5%)	8,287 (59.1%)
	*65-74*	7,300 (25.5%)	9,557 (30.3%)	3,813 (27.2%)
	*75+*	6,909 (24.1%)	6,472 (20.5%)	1,620 (11.6%)
				
gender	*male*	19,250 (67.1%)	20,924 (66.3%)	9,126 (65.1%)
	*female*	9,430 (32.9%)	10,621 (33.7%)	4,903 (35.0%)
				
income quintile*	*1*	5,723 (20.0%)	6,146 (19.5%)	2,418 (17.2%)
	*2*	6,053 (21.1%)	6,549 (20.8%)	2,755 (19.6%)
	*3*	5,893 (20.6%)	6,394 (20.3%)	2,889 (20.6%)
	*4*	5,685 (19.8%)	6,447 (20.4%)	3,071 (21.9%)
	*5*	5,326 (18.6%)	6,009 (19.1%)	2,896 (20.6%)
				
wait time	*mean +/- sd*	2.4 +/- 10.4	13.1 +/- 25.5	17.3 +/- 21.7

sd refers to standard deviation.

*Income quintile 1 is the lowest income category

### Impact of Urgency Scale, Gender, Age and SES

In Table 2, the results for proportional hazard models evaluating wait time to angiography are shown for each of the urgency categories, with larger HRs indicating shorter wait times. Within each category, the urgency score was a statistically significant predictor of time to angiography (HR for urgent patients 1.61, 95% CI 1.55-1.67; HR for semi-urgent patients 1.55, 95% CI 1.44-1.68; HR for elective patients 1.13, 95% CI 1.08-1.18). For urgent patients, each one unit increase in patient urgency corresponded to a 38% shorter wait time (Table 2)(Additional file 2 for calculation). For semi-urgent patients and elective patients, the improvements in wait time with greater patient urgency were of a smaller magnitude at 35% and 12% respectively with each unit change in the urgency score (Table 2).

**Table 2:** Relationship of Urgency Score, Age, Gender and SES to Wait Times for Coronary Angiography

Variable	Urgent (28,680)	Semi-Urgent (31,545)	Elective (14,029)
	HR (95% CI)		
Urgency Score*	1.61 (1.55-1.67)	1.55 (1.44-1.68)	1.13 (1.08-1.18)
20-39 yr male^†^	0.95 (0.83-1.09)	0.91 (0.71-1.17)	1.26 (0.98-1.62)
40-64 yr female^†^	0.77 (0.68-0.87)	0.70 (0.57-0.87)	1.11 (0.90-1.38)
40-64 yr male^†^	0.81 (0.72-0.91)	0.72 (0.58-0.89)	1.13 (0.91-1.39)
65-74 yr female^†^	0.70 (0.62-0.79)	0.68 (0.55-0.84)	1.12 (0.90-1.39)
65-74 male^†^	0.71 (0.63-0.80)	0.69 (0.56-0.85)	1.11 (0.90-1.37)
> 75 yr female^†^	0.68 (0.60-0.76)	0.72 (0.58-0.89)	1.19 (0.95-1.49)
> 75 yr male^†^	0.67 (0.59-0.76)	0.71 (0.57-0.87)	1.09 (0.88-1.36)
Income quintile 2**	1.02 (0.99-1.05)	1.02 (0.98-1.05)	1.06 (0.96-1.10)
Income quintile 3**	1.01 (0.98-1.04)	1.01 (0.98-1.05)	1.04 (0.98-1.11)
Income quintile 4**	1.02 (0.98-1.05)	1.00 (0.96-1.04)	1.05 (0.98-1.12)
Income quintile 5**	1.02 (0.98-1.05)	1.02 (0.98-1.07)	1.06 (0.97-1.15)

*Continuous scale from 1 to 7, with 7 as the most urgent. Hazard ratios are for each 1 unit change in the urgency score

^† ^Compared to reference of 20-39 yr female

** compared to income quintile 1, which is lowest income category.

HR is hazard ratio - HR greater than 1 indicates shorter wait time; CI is confidence interval.

The impact of age was most pronounced in the urgent and semi-urgent patients (Table 2). For example in urgent patients, when compared to 20-39 year old females, older males and females had substantially longer wait times (HR of 0.68 (95% CI 0.60-0.76) for females >75 yr and HR of 0.67 (95% CI 0.59-0.76) for males >75 years). In contrast, there was minimal difference in wait times between age and gender groups in the elective patients. SES did not appear to have a significant impact on wait-times after adjusting for urgency score and age in any of the urgency categories (Table 2).

### Regional Variation

**Figure 2:**
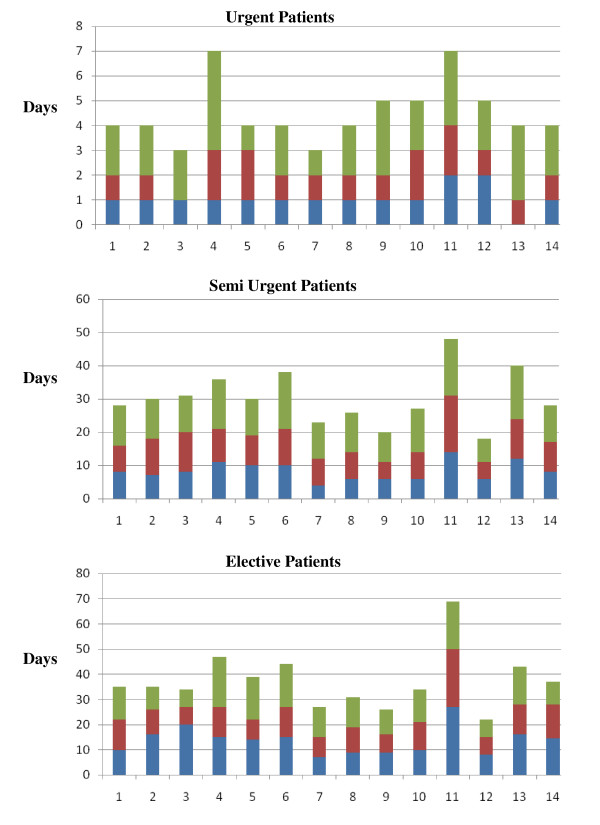
**Wait Times (Days) for Coronary Angiography per LHIN across Urgency Strata**. Green Bar 90 percentile. Red Bar 75 percentile. Blue Bar Median. 1 Erie St. Clair. 2 South West. 3 Waterloo Wellington. 4 Hamilton Niagara Haldimand Brant. 5 Central West. 6 Mississauga Halton. 7 Toronto Central. 8 Central. 9 Central East. 10 South East. 11 Champlain. 12 North Simcoe Muskoka.

The crude wait times across LHINs for the three urgency categories are shown in Figure 2. As wait times had a skewed distribution, median wait times and the 75^th ^and 90^th ^percentiles are shown. Although median wait times in urgent patients across LHINs were comparable, the percentage of urgent cases with wait times greater than the RMWT ranged from 14.2% (Toronto Central) to 35.8% (Champlain) (Table 3). The range of wait-times was greater in the semi-urgent and elective categories (Figure 2). For semi-urgent patients, the shortest median wait time was 10 days less than the longest median wait time; for elective patients, this difference was 20 days. A significant proportion of patients had wait times greater than the RMWT for both these categories. After adjusting for age-gender, SES and urgency score, there remained a statistical significant difference in wait times between LHINs (Table 3).

**Table 3:** Wait Times across Regions

	Urgent (N = 28,680)		Semi-Urgent (N = 31,545)		Elective (N = 14,029)	
	
Region	Above RMWT (%)	Hazard Ratio	Above RMWT (%)	Hazard Ratio	Above RMWT (%)	Hazard Ratio
Central Toronto	14.2	ref	28.5	ref	5.9	ref
Central	18.4	0.90 (0.82-0.98)	33.6	0.94 (0.80-1.10)	10.3	0.83 (0.67-1.04)
Central East	18.0	0.89 (0.82-0.97)	26.5	1.06 (0.91-1.24)	4.2	0.94 (0.79-1.13)
Central West	21.6	0.76 (0.70-0.82)	46.6	0.73 (0.61-0.87)	12.5	0.63 (0.51-0.78)
Champlain	35.8	0.62 (0.56-0.69)	58.2	0.50 (0.42-0.59)	38.4	0.31 (0.26-0.38)
Erie St Clair	17.6	0.81 (0.73-0.91)	41.7	0.76 (0.62-0.92)	15.0	0.64 (0.50-0.82)
Hamilton Niagara Haldimand Brant	32.9	0.65 (0.58-0.74)	53.4	0.58 (0.49-0.70)	19.7	0.50 (0.39-0.63)
Mississauga Halton	21.6	0.83 (0.73-0.93)	49.3	0.66 (0.56-0.78)	15.8	0.56 (0.46-0.67)
North East	15.8	0.99 (0.87-1.13)	53.8	0.60 (0.49-0.74)	22.8	0.52 (0.42-0.64)
North Simcoe Muskoka	27.6	0.76 (0.70-0.84)	27.7	1.13 (0.97-1.31)	5.2	1.08 (0.91-1.29)
North West	10.1	1.10 (0.95-1.27)	43.5	0.84 (0.69-1.02)	14.1	0.62 (0.50-0.76)
South East	24.1	0.82 (0.68-0.98)	33.4	0.89 (0.73-1.07)	12.6	0.70 (0.56-0.88)
South West	20.2	0.83 (0.73-0.95)	44.2	0.75 (0.61-0.92)	18.6	0.59 (0.48-0.72)
Waterloo Wellington	9.5	1.11 (0.97-1.27)	46.4	0.76 (0.63-0.93)	17.8	0.55 (0.45-0.67)

RMWT: recommended maximum waiting time. See Table 1 for abbreviations

### Impact of Supply Measures

In semi-urgent and elective patients, cath lab supply had a significant impact on residual waits times after accounting for urgency score and patient demographics (Table 4). With an increased allocation of one cath lab per 100,000 persons, there was a reduction in wait times of 50% (HR 2.01; 95% CI 1.33-3.04), and 64% (HR 2.74; 95% CI 1.60-4.69) for semi-urgent and elective patients respectively. The hazard ratio in urgent patients was 1.47 with a 95% CI of 0.98 to 2.21; however this did not reach statistical significance.

**Table 4:** Relationship of Supply Measures to Wait Times for Coronary Angiography

Variable	Urgent (28,680)	Semi-Urgent (31,545)	Elective (14,029)
	HR (95% CI)		
Cath Lab supply*	1.47 (0.98-2.21)	2.01 (1.33-3.04)	2.74 (1.60-4.69)
Invasive Cardiologist supply*	1.20 (1.14-1.26)	1.00 (0.93-1.08)	1.02 (0.95-1.10)
GP supply^†^	0.95 (0.91-0.98)	1.10 (1.04-1.16)	1.12 (1.05-1.19)

*Cath Lab, Invasive Cardiologist, supplies are per 100,000 persons of LHIN population.

†GP supply is per 10,000 persons of LHIN population.

See Table 1 for abbreviations.

The impact of invasive cardiologist supply was restricted to urgent patients, with a wait time reduction of 17% with an increased allocation of one invasive cardiologist per 100,000 persons (HR 1.20; 95% CI 1.14-1.26) (Table 4). In contrast, the impact of GP supply was confined to less urgent patients, with reductions of 9% and 11% seen in semi-urgent and elective patients respectively with each 1/10,000 persons increase in GP allocation (HR 1.10, 95% CI 1.04-1.16 in semi-urgent; HR 1.12, 95% CI 1.05-1.19 in elective patients).

## Discussion

This study assessed the relationship between demand-side factors, such as age, gender, SES and clinical need, and wait times for coronary angiography in a cohort of 74,254 patients. We found that a structured urgency rating system, reflecting clinical need, was effective in appropriately triaging patients waiting for coronary angiography with more urgent patients treated more rapidly. Nonetheless, there remained wide regional variations in wait-times. Although supply-side factors had an important impact on residual wait-times, there was differential role of cath lab, invasive cardiologist and GP supply depending on patient urgency. The most consistent contribution to this geographic variation was cath lab supply, with the impact of invasive cardiologist supply confined to urgent patients and that of GP supply confined to semi-urgent and elective patients. Our findings suggest that regional disparities in coronary angiography wait times may be addressed by initiatives aimed at improving both the supply of procedural facilities and access to both specialty and GP care.

Currently, most jurisdictions use implicit judgment in managing wait lists for coronary angiography[17]. Accordingly, there is the potential for non-clinical factors, such as SES having an inappropriate impact on patient triaging. The urgency score incorporates clinical determinants of patient need and has been validated against physician judgment and adverse events[18]. As such, when used as an explicit structured wait-time management tool to aid the appropriate triaging of patients, the urgency score may reduce previously documented access disparities across age, SES and geographic location.

We determined that the urgency score was a strong predictor of wait-times for angiography. Nonetheless, other variables continued to impact wait-times. For example, the effect of age was most pronounced in urgent patients, with older patients having delays to angiography despite being at the greatest risk and therefore having the potential for the greatest absolute risk reduction with an invasive strategy. Our findings reinforce the risk-treatment paradox that has been identified in multiple areas of medicine, including invasive cardiovascular care[19,20].

In contrast, SES differences in wait times were minimal, suggesting that a structured wait time management system has been successful in reducing access disparities for coronary angiography. Importantly, our study diverges from findings in previous studies[21]. Prior studies have shown that patients in lower socio-economic strata have a higher prevalence of coronary risk factors, and therefore would potentially be of higher urgency[22]. In our analysis, by adjusting for urgency score prior to SES, we may have mitigated much of the impact of SES. This is reinforced by the finding that SES had the greatest impact in elective patients, in whom clinical risk would be lower. Importantly, our analysis is limited by the absence of patient level data on income and SES, requiring postal codes instead to derive an income quintile. As such, given the risk for ecological fallacy, we cannot exclude the possibility that SES did play a demand-side role on wait times.

Despite accounting for patient urgency, there remained significant regional differences in wait times to coronary angiography. Although absolute differences in median wait times between LHINs were modest, there were substantial differences in the proportion of patients who were treated within RMWT. Previous work from the CCN registry suggests that, irrespective of urgency strata, the majority of all waiting-time deaths for angiography occur after the RMWT; moreover, had these patients been treated within the RMWT, an estimated 18.5 deaths per 10,000 patients may have been averted[23]. In our cohort from 2005-06, this translates to 15 potentially avoidable wait-time deaths across the three urgency groups.

In our analysis, cath lab supply had the most pronounced and consistent impact on regional differences in wait-times. The impact of procedural capacity was greatest in elective patients. This suggests that in regions with limited procedural capacity, there is appropriate prioritization of urgent patients, which in turn will amplify the delays for more elective angiograms. Cath lab supply is downstream from the CCN referral and therefore, predictably would have a direct impact on the delay to angiography.

Both invasive cardiology and GP supply had relatively modest impact on wait-times. Moreover, the impact of invasive cardiologists supply was restricted to urgent patients while that of GP supply was in less sick patients. A potential explanation for this observation is that urgent patients would more likely enter the health care system via a hospital admission and therefore require access to a specialist. In contrast, the primary care physician would more likely serve as the point of entry into the health care system for elective patients. As wait times are calculated from the time of CCN referral to the procedure, the impact of GP supply on time to cath is indirect. This impact may occur through greater patient advocacy, with the GP acting to ensure their patients receive prompt treatment. Therefore, we hypothesize that GP supply corresponds with the overall efficiency of health care delivery within the region.

These differential impacts underscore the inter-relationship between these components of care. As such, our data suggests that if greater equity in access to angiography across regions is to be achieved, initiatives should be directed at improving all supply components. An important caveat to our conclusions is the absence of a direct measure of the appropriateness of coronary angiography. The appropriateness for coronary angiography should be a reflection of the clinical need for invasive cardiac investigation. Although the urgency score captures some elements of clinical need, it is not an ideal proxy for appropriateness, because it fails to incorporate other important factors such as frailty, which may 'appropriately' delay angiography. The inability to fully evaluate procedural appropriateness into our analysis highlights the need for a more direct metric of this critical demand-supply factor.

Several additional limitations of our study merit consideration. First, this study used data from 2005 to 2006. Since that time, coronary angiography facilities in Ontario have increased substantially, and we would anticipate overall wait times have reduced. However, concurrent to this increase in supply, the indications for coronary angiography have expanded, especially for patients hospitalized with acute coronary syndromes[24,25]. As a sensitivity analysis, we repeated our analysis using data from 2001-2004. As seen in Additional file 1, we found similar results using this earlier cohort, with both cath lab and physician supply playing a larger role. Indeed, as procedural capacity has improved in recent years, physician factors have played a lesser role in residual wait times. Therefore, we anticipate that our conclusions will apply to contemporary practice, although the magnitude may vary.

Second, the use of the urgency score may vary from institution to institution. Although some may use the score itself to triage, others may use it as a monitoring tool to assess if implicit triage is appropriate. Nonetheless, given the allocation of RMWT to urgency scores by the CCN and ongoing surveillance of institutional adherence to these guidelines, the urgency score provides a structured framework for wait time management.

Third, we performed a complete case analysis, assuming that the 12% of patients with missing data were missing completely at random (MCAR). In Additional file 3, we examined the characteristics of patients with complete data and those with missing data elements, and found the cohorts were reasonably matched. However, there were differences in the urgency score and wait times between the complete case cohort and those with missing data (urgency score of 4.1 for missing data versus 3.9 for complete case cohort; wait-time of 12.6 days for the missing data versus 9.7 days in the complete case cohort). In addition, it appeared that the missing data was not evenly distributed between regions. As such, we cannot rule out the possibility that the pattern of missing data is contingent on the other available observed parameters such as regional supply or on any unobserved characteristics of the patients themselves.

Finally, our study only addresses the differences in access to angiography of patients who have already been assessed by a physician and thereby referred for a diagnostic procedure. Arguably, a substantial burden of disease exists in the proportion of the population who has no access to physicians, and therefore never evaluated nor referred. Therefore, it is likely that the impact of supply measures on regional access disparities, especially that of GP supply, is in fact greater than we have described.

## Conclusions

In summary, using a large cohort of patients who underwent coronary angiography, we found that an explicit urgency rating scale was effective in triaging patients based on clinical need and thereby minimized the impact of other demand-side factors such as SES. Nonetheless, wide variations in regional access to care remain which are in part mediated by geographic variations in supply-side measures.

## Competing interests

**DAA **is the Chief Scientific Officer and consultant for INTER_x_VENT Canada (PrevCan™), a therapeutic lifestyle and disease management program. None of the other authors have any conflict of interests to disclose.

## Authors' contributions

**HCW**: Conception, design, analysis and interpretation of data; drafting of manuscript; final approval of manuscript submitted. **TAS**: Conception, design, analysis and interpretation of data; revising of manuscript; final approval of manuscript submitted; **AC**: Analysis and interpretation of data; revising of manuscript; final approval of manuscript submitted. **MKN**: Interpretation of data; revising of manuscript; final approval of manuscript submitted. **DAA**: Conception, design, analysis and interpretation of data; revising of manuscript; final approval of manuscript submitted.

## About the authors

HCW:

Room A134c, 2075 Bayview Avenue,

Sunnybrook Health Sciences Center, Toronto, ON M4N 3M5

Office phone: (416) 480-6100 ext 4527 Email: wijeysundera@sympatico.ca

TAS:

Room G106, 2075 Bayview Avenue,

Institute for Clinical Evaluative Sciences, Toronto, ON M4N 3M5

Office phone: (416) 480-6100 ext 3928 Email: stukel@ices.on.ca

AC:

2075 Bayview Avenue,

Institute for Clinical Evaluative Sciences, Toronto, ON M4N 3M5

Office phone: (416) 480-4055 ext 3076 Email: alice.chong@ices.on.ca

MKN:

McMaster Clinic, Room 263,

237 Barton Street East, Hamilton, ON

Office phone: 905-521-2100 ext 44839 Email:nataraja@mcmaster.ca

DAA:

Room G-106, 2075 Bayview Avenue,

Institute for Clinical Evaluative Sciences, Toronto, ON M4N 3M5

Office phone: (416) 480-5838 Email: david.alter@ices.on.ca

## Pre-publication history

The pre-publication history for this paper can be accessed here:

http://www.biomedcentral.com/1472-6963/10/5/prepub

## Supplementary Material

Additional file 1 **Appendix 1**. Relationship of supply measures to wait times for coronary angiography based on 2001-2006 data. Click here for file

Additional file 2 **Appendix 2**. Conversion of hazard ratio to percentage change. Click here for file

Additional file 3 **Appendix 3**. Comparison of complete-case and missing data cohorts. Click here for file
