# Supplementary material for: Impact of clinical urgency, physician supply and procedural capacity on regional variations in wait times for coronary angiography
Source: BMC Health Serv Res. 2010 Jan 5;10:5. doi: 10.1186/1472-6963-10-5 (PMC2826304; doi:10.1186/1472-6963-10-5)
Supplement: Additional file 1 — Appendix 1. Relationship of supply measures to wait times for coronary angiography based on 2001-2006 data. [file 1472-6963-10-5-S1.DOC]

**Appendix 1: Relationship of supply measures to wait times for coronary angiography based on 2001-2006 data**

| **Variable** | **2001-2002** | **2003-2004** | **2005-2006** |
| --- | --- | --- | --- |
|  | HR (95% CI) | | |
| **URGENT** | | | |
| Cath Lab supply^*^ | 2.95  (1.80-4.83) | 1.63  (0.96 -2.78) | 1.47  (0.98-2.21) |
| Invasive Cardiologist supply^*^ | 1.16  (1.10-1.23) | 1.18  (1.11-1.25) | 1.20  (1.14-1.26) |
| GP supply^†^ | 1.21  (1.16-1.26) | 1.11  (1.06-1.16) | 0.95  (0.91-0.98) |
| **SEMI-URGENT** | | | |
| Cath Lab supply^*^ | 3.17  (1.53-6.56) | 1.10  (0.66-1.82) | 2.01  (1.33-3.04) |
| Invasive Cardiologist supply^*^ | 1.01  (0.92-1.10) | 1.09  (1.00-1.17) | 1.00  (0.93-1.06) |
| GP supply^†^ | 1.27  (1.18-1.37) | 1.27  (1.19-1.36) | 1.10  (1.04-1.16) |
| **ELECTIVE** | | | |
| Cath Lab supply^*^ | 2.85  (1.18-6.90) | 1.09  (0.60-1.97) | 2.74  (1.60-4.69) |
| Invasive Cardiologist supply^*^ | 1.04  (0.94-1.15) | 1.08  (0.99-1.17) | 1.02  (0.95-1.10) |
| GP supply^†^ | 1.37  (1.25-1.51) | 1.35  (1.25-1.45) | 1.12  (1.05-1.19) |

**^*^**Cath Lab, Invasive Cardiologist, supplies are per 100,000 persons of LHIN population.

†GP supply is per 10,000 persons of LHIN population.

See Table 1 for abbreviations.
