# Supplementary material for: Impact of clinical urgency, physician supply and procedural capacity on regional variations in wait times for coronary angiography
Source: BMC Health Serv Res. 2010 Jan 5;10:5. doi: 10.1186/1472-6963-10-5 (PMC2826304; doi:10.1186/1472-6963-10-5)
Supplement: Additional file 2 — Appendix 2. Conversion of hazard ratio to percentage change. [file 1472-6963-10-5-S2.DOC]

**Appendix 2: Conversion of hazard ratio to percentage change**

In order to convert the Hazard Ratios (HR) found in the results section to a percentage decrease in wait time, the following equation is used:

% decrease in wait time = (1-1/HR)
