# Supplementary material for: Impact of clinical urgency, physician supply and procedural capacity on regional variations in wait times for coronary angiography
Source: BMC Health Serv Res. 2010 Jan 5;10:5. doi: 10.1186/1472-6963-10-5 (PMC2826304; doi:10.1186/1472-6963-10-5)
Supplement: Additional file 3 — Appendix 3. Comparison of complete-case and missing data cohorts. [file 1472-6963-10-5-S3.DOC]

**Appendix 3: Comparison of complete-case and missing data cohorts**

|  | | **Complete Case Cohort** | **Missing Data Cohort** | **Standardized Difference (%)^**^** |
| --- | --- | --- | --- | --- |
| Sample size |  | 74,254 | 10,594 |  |
| age | *mean ± sd* | 63.50 ± 11.92 | 63.38 ± 13.15 | 1% |
| gender | *male* | 49,300 (66.4%) | 6,736 (63.6%) | 6% |
| income quintile^*^ | *1* | 14,287 (19.2%) | 1,736 (19.0%) | 1% |
|  | *2* | 15,357 (20.7%) | 1,872 (20.5%) |  |
|  | *3* | 15,176 (20.4%) | 1,837 (20.1%) |  |
|  | *4* | 15,203 (20.5%) | 1,867 (20.5%) |  |
|  | *5* | 14,231 (19.2%) | 1,808 (19.8%) |  |
| Urgency rating score^†^ | *mean ± sd* | 3.9 ± 1.3 | 4.1 ± 1.3 | 17% |
| Wait time | *mean ± sd* | 9.7 ± 21.1 | 12.6 ± 23.2 | 13% |
| Region | *Erie St. Clair* | 4,208 (5.7%) | 486 (5.2%) | 19% |
|  | *South West* | 4,476 (6.0%) | 745 (8.0%) |  |
|  | *Waterloo Wellington* | 3,138 (4.2%) | 289 (3.1%) |  |
|  | *Hamilton Niagara Haldimand Brant* | 9,744 (13.1%) | 1,095 (11.7%) |  |
|  | *Central West* | 4,173 (5.6%) | 356 (3.8%) |  |
|  | *Mississauga Halton* | 5,401 (7.3%) | 498 (5.3%) |  |
|  | *Toronto Central* | 5,785 (7.8%) | 542 (5.8%) |  |
|  | *Central* | 8,313 (11.2%) | 807 (8.6%) |  |
|  | *Central East* | 9,555 (12.9%) | 1,110 (11.9%) |  |
|  | *South East* | 4,642 (6.3%) | 367 (3.9%) |  |
|  | *Champlain* | 5,829 (7.9%) | 1,120 (12.0%) |  |
|  | *North Simcoe Muskoka* | 2,877 (3.9%) | 319 (3.4%) |  |
|  | *North East* | 4,520 (6.1%) | 1,037 (11.1%) |  |
|  | *North West* | 1,593 (2.1%) | 579 (6.2%) |  |

sd refers to standard deviation.

^*^Income quintile 1 is the lowest income category

^†^Continuous scale from 1 to 7, with 7 as the most urgent

** Standardized difference is defined as the absolute difference between the mean of the complete case cohort and the mean of the missing data cohort, divided by the standard deviation of the entire cohort.
